# Associations between diet quality, demographics, health conditions and spice and herb intake of adults with chronic kidney disease

**DOI:** 10.1371/journal.pone.0298386

**Published:** 2024-03-07

**Authors:** Emily Hammer, Sofia Acevedo, Jeanette Mary Andrade

**Affiliations:** Food Science and Human Nutrition, University of Florida, Gainesville, Florida, United States of America; Universidad Autonoma de Chihuahua, MEXICO

## Abstract

**S**cant literature has been able to demonstrate an association between dietary habits and spice and herb consumption, especially for those who have chronic kidney disease. The objectives of this study were to 1) determine the frequency and quantity of spices and herbs consumed and 2) determine the associations between diet quality and its food components, demographics, and health conditions with spice and herb frequency and variety consumption of adults with chronic kidney disease. A cross-sectional online study was conducted with adults with various stages of chronic kidney disease (n = 71). Participants responded to an online demographic, diet and spice and herb questionnaire on RedCap. Diet quality was determined through the diet questionnaire. Descriptives, frequencies and Spearman correlations were conducted using SPSS v28 with a significance of p<0.05. Most participants were in chronic kidney disease stage 3 (42.3%) with a majority (98.6%) self-identifying as non-Hispanic white. On average, participants consumed black pepper more than once daily (47.9%) with the spice quantity at 5 g. The median diet quality score was 38.5 (range 31.5–48.5). Positive associations were identified with overall diet quality scores and certain spices such as basil (r = 0.33; p<0.01) and cinnamon (r = 0.37; p<0.002). Further associations were seen with food groups, self-identifying as white and health conditions with spice frequency and variety of spices and herbs consumed. Overall, positive associations were observed with diet quality and spice and herb intake, in which higher diet quality scores would indicate higher consumption of spices and herbs. Further research should focus on diet quality and spice and herb consumption in reducing progression of this disease.

## Introduction

Chronic Kidney Disease (CKD) is considered a low-grade chronic inflammatory progressive disorder that has five stages. Stage one is mild kidney damage with normal/high estimated glomerular filtration rate (eGFR) of >90 mL/min/1.73 m^2^ and stage five is considered kidney failure with eGFR of <15 mL/min/1.73 m^2^ [1]. CKD is often undiagnosed in the earlier stages due to lack of symptoms [2,3]. However, as the disease progresses, symptoms such as fatigue, pruritis, and depression occur, which can impact an individual’s quality of life by interfering with relationships, financial status, and mental health [4]. CKD affects about 9.1% of the worldwide population [5]and within the United States, 37 million individuals are diagnosed with this disease with one in three Americans being at risk for CKD [3]. Risk factors for CKD include hypertension, diabetes, being overweight, and poor self-management behaviors such as diet and physical activity [6,7].

One strategy to delay complications and progression of CKD is through diet [6,8]. Diet recommendations for adults with CKD are dependent on laboratory values, co-morbidities, and stage of CKD, which focuses on adequate intake of certain nutrients such as protein, sodium, and phosphorus [1]. Through a review, it was discovered that adults at end-stage kidney disease have a diet adherence of 31.5% [9] due to multiple factors which may include finances, fatigue and aversions to certain foods such as red meat [9–12]. Recent studies conducted in the US have observed that adults with CKD have an overall low diet quality, which is related to limited intake of whole grains, fruits, and vegetables [12–14]. Diet quality is a metric used to determine how close one’s diet is to the dietary guidelines based on food groups. Based on the Healthy Eating Index (HEI) 2015, a higher score indicates that a person is closely adhering to the dietary guidelines for Americans [15]. The diet quality score does not account for any spices and herbs used that may potentially aid in reducing progression of CKD. Further, the associations with diet quality and spice and herb consumption are inconsistent, in which the higher spice and herb intake may or may not yield higher diet quality [16–18]. This may ultimately affect the progression of a disease.

Spices and herbs have been used for medicinal purposes for centuries in various cultures [19,20]. Studies have shown that inflammation can be reduced, which improves renal and cardiac functions, if the individual consumes spices and herbs such as clove, garlic, ginger, turmeric, rosemary, and pepper due to the active components that are able to inhibit inflammatory pathways [19–23]. Currently, the amount and frequency of spice and herb intake among the CKD population is not known, nor if their dietary intake as quantified by diet quality has an association with spice and herb consumption. The objectives of this study were to 1) determine the frequency and quantity of spices and herbs consumed and 2) determine the associations between diet quality and its food components, demographics, and health conditions with spice and herb frequency and variety consumption of adults with CKD.

## Materials and methods

A cross-sectional online study was conducted from November 2022 –January 2023. Participants were recruited through Researchmatch [24] and CKD Support Facebook groups. For ResearchMatch, criteria were selected to only recruit participants who had self-identified as being diagnosed with CKD of any stage. The CKD Support Facebook groups that were targeted included “Kidney Disease / Failure Support Group” and “Chronic Kidney Disease (CKD) Support Forum”. This recruitment approach was adopted to attain a substantial and diverse sample size and that this approach was considered user-friendly and efficacious in enlisting eligible and competent participants. Adults who were > 18 years old, were able to read English, and had been diagnosed with CKD of any stage were qualified to participate. Prospective participants were screened online prior to completing it. They were asked if they were diagnosed with CKD. If prospective participants indicated they were not diagnosed with CKD, they were excluded from the study. Individuals who did not meet these criteria were excluded. An online RedCap survey on dietary and spice and herb intake was administered to the participants. A total of 78 participants initiated the survey with only 71 completing the entirety of the survey to be used in the analysis. The University of Florida Institutional Review Board (IRB) granted exemption for this study.

### Demographic and health conditions questionnaire

The demographic questionnaire included gender, age, race/ethnicity, self-reported stage of CKD and when diagnosed and additional health conditions/diseases. Participants were able to select multi-responses for additional health conditions/diseases. Participants were given the option to select none, do not know or prefer not to respond.

### Chronic Kidney Disease Short Food Frequency Questionnaire

The Chronic Kidney Disease Short Food Frequency Questionnaire (CKD-SFFQ) is a validated tool that assesses frequency of consuming 57 food and beverage items over 30-days for those with CKD [13]. As this instrument was validated in the US, portion sizes were displayed as imperial units for participants to approximately indicate the frequency of consuming foods and beverages. Responses included: daily, 1–2 servings weekly, 3–5 servings weekly, or rarely/never. These food items are then categorized into an overall diet quality and nine individual whole food components: total fruits (juices, canned, dried) and whole fruits (3 items), total vegetables (canned, fresh) (7 items), greens and beans (1 item), whole grains (3 items), refined grains (8 items), dairy (4 items), total protein foods (12 items), and seafood and plant proteins (3 items). The items within the whole food components are provided with an unweighted score of 0–2 and then summed for a total score range of 0–60 with a score of ≤20 indicating a low-quality diet, score of 21–39 indicating a diet that needs improvement and a score of ≥40 indicating a high-quality diet [13]. The relative validity of this instrument was compared with the Healthy-Eating Index– 2015 (HEI-2015) through the overall diet quality and nine individual whole food components and showed good agreement [13].

### Spice and herb consumption questionnaire

A 50-item validated spice and herb consumption questionnaire was administered that focused on the frequency and quantity of consuming 25 spices and herbs over 30-days [25,26]. Responses for the frequency of consuming spices were: 6 or more times per day, 4–5 times per day, 2–3 times per day, 1 time per day, 5–6 times per week, 2–4 times per week, 1 time per week, 1–3 times per month, or never/less than once per month. Participants were then asked to identify the amount of each spice and herb that they consumed. Participants were given the following response options without pictorial figures for metric amounts: more than 5 g, 5 g, less than 5 g, do not measure, or never. Total spice and herb variety was summed based on the spices and herbs consumed once or more daily and those spices and herbs consumed less than once daily.

### Statistical analysis

Frequency counts and percentages were tabulated for demographic variables, dietary intake, and spice and herb consumption. Mean ± standard deviation or median (interquartile range) was used for presenting the data with normal or non-normal distribution. Spearman correlations were conducted to determine associations between diet quality and its food components, demographics, and health conditions with spice and herb frequency and variety consumption. Based on the number of participants identifying the various stages of CKD, sub-group analyses were conducted by stratifying to two groups: early stages of CKD (1–2) or later stages (3–5). Participants who indicated ‘do not know’ the CKD stage (n = 6) were excluded from any additional sub-group analyses. Mann-Whitney U was conducted to detect differences in earlier stages of CKD compared to later stages of CKD with frequency and quantity of spices consumed. Significance was determined with a p-value of ≤ 0.05 using SPSS v28.

## Results and discussion

Of the 71 participants, the majority (77.5%; n = 51) were female, at least 60 years of age or older (56.4%; n = 40), and self-identified as white, non-Hispanic (78.9%; n = 56). Participants self-identified that they were in CKD stage 3 (41.4%; n = 29) with 46.4% (n = 33) indicating that they were diagnosed with CKD more than 5 years ago. The most common condition that participants had in conjunction with CKD was high blood pressure (66.2%; n = 47). Based on the variety of spices and herbs that participants consumed once or more daily or less than once daily, the categories were condensed to once or more daily or less than once daily, excluding those spices and herbs never consumed. Total average spice and herb variety for participants that consumed them more than once daily was 2.96 (±4.3) and total average spice and herb variety for participants that consumed them less than once daily was 11.2 (±6.4) in Table 1.

**Table 1:** Participants’ demographics, health conditions and total spice and herb variety consumed (n = 71).

	N (%)
Gender	
Male	15 (21.1%)
Female	55 (77.5%)
Other	1 (1.4%)
Age Range	
25–29	1 (1.4%)
30–49	20 (28.2%)
50–59	10 (14.0%)
60–69	20 (28.2%)
70+	20 (28.2%)
Race	
Asian / Native American	1 (1.4%)
Black	11 (15.5%)
White	56 (78.9%)
Multi-racial	3 (4.2%)
Ethnicity	
Hispanic	1 (1.4%)
Non-Hispanic	70 (98.6%)
Stage of CKD	
1 /2	11 (15.5%)
3	30 (42.3%)
4	8 (11.4%)
5	6 (8.6%)
Do not know	16 (22.2%)
Years with CKD	
< 1 year	17 (23.9%)
1–2 years	8 (11.4%)
3–4 years	13 (18.3%)
5 years or longer	33 (46.4%)
Chronic conditions	
Cancer	4 (5.6%)
Depression	22 (31.0%)
Diabetes	21 (30.0%)
Diverticulosis / Diverticulitis	6 (8.4%)
Gastric reflux	17 (23.9%)
Heart disease	13 (18.3%)
Hypertension	47 (66.2%)
Irritable bowel	15 (21.1%)
Liver issues	7 (9.9%)
Nausea / Vomiting	8 (11.3%)
Average total spice and herb variety of consuming at least once daily or more	Average: 2.96 g Standard deviation: (±4.3)
Average total spice and herb variety of consuming less than once daily to once monthly	Average: 11.2 g Standard deviation: (±6.4)

### Dietary intake

On average, the median diet quality score was 38.8 (range between 31.5 to 48.5) out of 60 with lower scores seen in the food groups–whole fruits and total fruits, whole grains, greens and beans, seafood, and plant proteins. Higher food group scores were observed in refined grains and total proteins as seen in Table 2.

**Table 2:** Diet quality scores (n = 71).

Components	Scoring range (min–max)	Median (range)
Overall diet quality	0–60	38.5 (31.5–48.5)
Whole grains	0–6	1.5 (0–6)
Refined grains	0–14	8.5 (1–14)
Greens and beans	0–2	1 (0–2)
Total vegetables	0–7	4 (2–7)
Whole fruit / Total fruit	0–3	3 (1–3)
Seafood and plant proteins	0–5	2.5 (0–4.5)
Total proteins	0–15	10.5 (6–13)
Dairy	0–8	3 (1–6)

Based on the actual CKD-FFQ of the food and beverage items, participants consumed coffee and water daily (51% and 83%, respectively), vegetables, raw fruits, and olive oil at 3 or more times weekly (58%, 45%, and 51%, respectively), and baked or grilled chicken and eggs prepared with no fat at least 1–2 times weekly (56% and 34%, respectively) as seen in Table 3.

**Table 3:** Frequency of food and beverage intake (n = 71).

Food / beverage items	Rarely / Never	1–2 Portions weekly	3–5 Portions weekly	Daily
	N (%)			
Whole grains				
Whole grain/ Wheat/ Brown bread	27 (38.0%)	24 (33.8%)	11 (15.5%)	9 (12.7%)
Whole grain/ Brown: pasta and rice	45 (63.4%)	20 (28.2%)	3 (4.2%)	3 (4.2%)
Whole grain breakfast cereals	45 (63.4%)	20 (28.2%)	3 (4.2%)	3 (4.2%)
Refined grains				
White bread	39 (54.9%)	14 (19.7%)	10 (14.1%)	8 (11.3%)
White pasta and rice	32 (45.1%)	26 (36.6%)	10 (14.1%)	3 (4.2%)
Non-whole grain breakfast cereals	57 (80.3%)	10 (14.1%)	4 (5.6%)	0
Soup–broth based	37 (52.1%)	24 (33.8%)	8 (11.3%)	2 (2.8%)
Soup–cream based	55 (77.5%)	11 (15.5%)	3 (4.2%)	2 (2.8%)
Processed non homemade, Sandwiches, burgers, burritos, tamales, enchiladas, tacos	50 (70.5%)	16 (22.5%)	3 (4.2%)	2 (2.8%)
Salty snacks	39 (54.9%)	22 (31.0%)	8 (11.3%)	2 (2.8%)
Sweet cakes and snacks, chocolate and pastries	29 (40.8%)	26 (36.6%)	12 (16.9%)	4 (5.6%)
Plant proteins/ Vegetables				
Legumes, beans	19 (26.8%)	32 (45.1%)	16 (22.5%)	4 (5.6%)
Potatoes baked	43 (60.6%)	22 (31.0%)	5 (7.0%)	1 (1.4%)
Potatoes roasted	49 (69.0%)	16 (22.5%)	5 (7.0%)	1 (1.4%)
Potatoes mashed prepared with butter and milk	54 (76.1%)	12 (16.9%)	4 (5.6%)	1 (1.4%)
Potatoes mashed prepared with water	61 (85.9%)	7 (9.9%)	2 (2.8%)	1 (1.4%)
French fries	49 (69.0%)	18 (25.4%)	2 (2.8%)	2 (2.8%)
Tomatoes: raw, cooked	20 (28.1%)	31 (43.7%)	18 (25.4%)	2 (2.8%)
Vegetables (not counting potatoes): raw, cooked with no oil or salt	12 (16.9%)	18 (25.4%)	26 (36.6%)	15 (21.1%)
Whole fruit / Total fruit				
Fruit raw	13 (18.3%)	26 (36.6%)	14 (19.7%)	18 (25.4%)
Canned fruit	52 (73.3%)	11 (15.5%)	5 (7.0%)	3 (4.2%)
Dried fruit	55 (77.4%)	9 (12.7%)	5 (7.0%)	2 (2.8%)
Seafood / Total proteins				
Pizza, lasagna, quiche	44 (62.0%)	20 (28.2%)	5 (7.0%)	2 (2.8%)
Homemade; Sandwiches, burgers, burritos, tamales, enchiladas, tacos	22 (31.0%)	28 (39.4%)	15 (21.1%)	6 (8.5%)
Fish: fried, breaded	53 (74.7%)	15 (21.1%)	2 (2.8%)	1 (1.4%)
Fish: baked or grilled	35 (49.3%)	30 (42.3%)	4 (5.6%)	2 (2.8%)
Seafood (other than fish; tuna, sardines)	53 (74.7%)	12 (16.9%)	5 (7.0%)	1 (1.4%)
Chicken: fried, breaded	57 (80.3%)	9 (12.7%)	4 (5.6%)	1 (1.4%)
Chicken: baked or grilled	15 (21.1%)	40 (56.3%)	14 (19.7%)	2 (2.8%)
Red meat (beef, lamb, deer, camel): fried, Breaded	54 (76.1%)	13 (18.3%)	3 (4.2%)	1 (1.4%)
Red meat (beef, lamb, deer, camel): baked or Grilled	38 (53.5%)	26 (36.6%)	5 (7.0%)	2 (2.8%)
Pork: fried, breaded	63 (88.8%)	6 (8.5%)	1 (1.4%)	1 (1.4%)
Pork: baked or grilled	47 (66.2%)	21 (30.0%)	2 (2.8%)	1 (1.4%)
Sausages or other processed meats	47 (66.2%)	18 (25.4%)	4 (5.6%)	2 (2.8%)
Eggs: boiled, scrambled, omelet prepared with Fat	32 (45.1%)	25 (35.2%)	9 (12.7%)	5 (7.0%)
Eggs: boiled, scrambled, omelet prepared without fat	32 (45.1%)	22 (31.0%)	12 (16.9%)	5 (7.0%)
Dairy				
Milk, Yogurt: 2% or skim	39 (54.9%)	15 (21.1%)	6 (8.5%)	11 (15.5%)
Milk, Yogurt: Whole or full fat	48 (67.5%)	11 (15.5%)	6 (8.5%)	6 (8.5%)
Cheese soft (Mozzarella, Blue cheese, Feta)	33 (46.5%)	25 (35.2%)	12 (16.9%)	1 (1.4%)
Cheese hard (Cheddar, Swiss)	24 (33.8%)	33 (46.5%)	11 (15.5%)	3 (4.2%)
Condiments / Oils				
Cheese spread: cream	46 (65.8%)	21 (30.0%)	1 (1.4%)	2 (2.8%)
Butter, sour cream	27 (38.0%)	16 (22.5%)	16 (22.5%)	12 (16.9%)
Margarine, mayonnaise	40 (56.4%)	16 (22.5%)	10 (14.1%)	5 (7.0%)
Mustard, salty condiments	30 (42.3%)	29 (40.8%)	10 (14.1%)	2 (2.8%)
Ketchup, sweetened sauces (duck sauce)	45 (63.4%)	19 (26.8%)	6 (8.5%)	1 (1.4%)
Olive oil	13 (18.3%)	22 (31.0%)	24 (33.8%)	12 (16.9%)
Rapeseed oil, walnut oil, mixed oil	64 (90.2%)	1 (1.4%)	5 (7.0%)	1 (1.4%)
Canola oil, sunflower oil	54 (76.1%)	11 (15.5%)	4 (5.6%)	2 (2.8%)
Coconut oil	63 (88.8%)	4 (5.6%)	3 (4.2%)	1 (1.4%)
Sugar white, brown, cane	45 (63.4%)	13 (18.3%)	5 (7.0%)	8 (11.3%)
Artificial sweeteners: Sweet’n low, Splenda, Equal, Stevia, etc	47 (66.2%)	10 (14.1%)	4 (5.6%)	10 (14.1%)
Beverages				
Coffee	22 (31.0%)	7 (9.9%)	4 (5.6%)	38 (53.5%)
Freshly brewed tea or herb teas	33 (46.5%)	15 (21.1%)	8 (11.3%)	15 (21.1%)
Canned/ bottled tea or herb teas	58 (81.7%)	9 (12.7%)	2 (2.8%)	2 (2.8%)
Sweetened beverages (soda)	59 (83.1%)	4 (5.6%)	6 (8.5%)	2 (2.8%)
Artificially sweetened beverages (diet soda)	47 (66.2%)	10 (14.1%)	4 (5.6%)	10 (14.1%)
Water	4 (5.6%)	3 (4.2%)	4 (5.6%)	60 (84.6%)
Wine	56 (78.9%)	6 (8.5%)	5 (7.0%)	4 (5.6%)
Beer	55 (77.4%)	8 (11.3%)	7 (9.9%)	1 (1.4%)
Other alcoholic beverages	58 (81.7%)	8 (11.3%)	4 (5.6%)	1 (1.4%)

### Spice and herb intake

The questionnaire used was initially validated on healthy adults and those with cancer. Therefore, through a reliability analysis with the responses, the questionnaire was deemed acceptable with a Cronbach alpha of 0.98. On average, participants consumed black pepper and garlic one or more times daily at 47.9% and 31.0%, respectively, with at least 5 g consumed at these times. Most participants rarely or never consumed Adobo seasoning (blend of garlic, oregano, pepper, paprika, and onion powder) (87.1%), curry leaf and fennel, both at 71.8%. When displaying the results, categories were condensed due to the limited frequency of spice and herb consumption in Table 4.

**Table 4:** Frequency and quantity of spice intake (n = 71).

Frequency						Quantity			
	Never or less than once monthly	1–3 times monthly	Once weekly	2–6 times weekly	1 or more times daily	Never/ do not measure	less than <5 g	5 g	more than >5 g
Spices	N (%)				N (%)				
Adobo seasoning	58 (81.7%)	6 (8.5%)	3 (4.2%)	2 (2.8%)	2 (2.8%)	54 (76.1%)	6 (8.5%)	7 (9.8%)	4 (5.6%)
Basil	21 (29.7%)	14 (19.7%)	14 (19.7%)	14 (19.7%)	8 (11.3%)	17 (23.9%)	16 (22.5%)	28 (39.4%)	10 (14.1%)
Black pepper	13 (18.4%)	2 (2.8%)	5 (7.0%)	17 (23.9%)	34 (47.9%)	10 (14.1%)	14 (19.7%)	35 (49.3%)	12 (16.9%)
Cajun seasoning	42 (59.2%)	5 (7.0%)	11 (15.5%)	7 (9.9%)	6 (8.5%)	42 (59.2%)	6 (8.5%)	19 (26.8%)	1 (1.4%)
Chili pepper	31 (43.7%)	10 (14.1%)	9 (12.7%)	14 (19.7%)	7 (9.9%)	28 (39.4%)	13 (18.4%)	22 (31.0%)	8 (11.3%)
Cilantro	35 (49.3%)	13 (18.4%)	7 (9.9%)	9 (12.7%)	7 (9.9%)	39 (54.9%)	10 (14.1%)	19 (26.8%)	3 (4.2%)
Cinnamon	20 (28.1%)	10 (14.1%)	11 (15.5%)	18 (25.4%)	12 (16.9%)	18 (25.4%)	13 (18.4%)	32 (45.1%)	8 (11.3%)
Cloves	39 (54.8%)	13 (18.4%)	6 (8.5%)	8 (11.3%)	5 (7.0%)	44 (61.8%)	13 (18.4%)	13 (18.4%)	1 (1.4%)
Coriander	48 (67.7%)	7 (9.9%)	5 (7.0%)	5 (7.0%)	6 (8.5%)	50 (70.4%)	4 (5.6%)	16 (22.5%)	1 (1.4%)
Cumin	32 (45.1%)	10 (14.1%)	11 (15.5%)	14 (19.7%)	4 (5.6%)	33 (46.5%)	11 (15.5%)	23 (32.4%)	4 (5.6%)
Curry leaf	51 (71.8%)	7 (9.9%)	3 (4.2%)	6 (8.5%)	4 (5.6%)	54 (76.1%)	4 (5.6%)	12 (16.9%)	1 (1.4%)
Curry seasoning	37 (52.1%)	10 (14.1%)	13 (18.4%)	6 (8.5%)	5 (7.0%)	38 (53.5%)	10 (14.1%)	18 (25.4%)	5 (7.0%)
Fennel	51 (71.8%)	7 (9.9%)	4 (5.6%)	3 (4.2%)	5 (7.0%)	54 (76.1%)	4 (5.6%)	11 (15.5%)	2 (2.8%)
Garlic	8 (11.3%)	8 (11.3%)	8 (11.3%)	25 (35.2%)	22 (31.0%)	9 (12.7%)	6 (8.5%)	32 (45.1%)	24 (33.8%)
Ginger	25 (35.2%)	16 (22.5%)	15 (21.1%)	4 (5.6%)	11 (15.5%)	32 (45.1%)	13 (18.4%)	18 (25.4%)	8 (11.3%)
Greek seasoning	46 (64.8%)	12 (19.6%)	3 (4.2%)	6 (8.5%)	4 (5.6%)	50 (70.4%)	5 (7.0%)	15 (21.1%)	1 (1.4%)
Italian seasoning	15 (21.1%)	17 (23.9%)	10 (14.1%)	24 (33.8%)	5 (7.0%)	18 (25.4%)	16 (22.5%)	27 (38.0%)	10 (14.1%)
Mint	38 (53.5%)	19 (26.8%)	2 (2.8%)	8 (11.3%)	4 (5.6%)	43 (60.6%)	7 (9.8%)	20 (28.1%)	1 (1.4%)
Oregano	18 (25.4%)	14 (19.7%)	11 (15.5%)	21 (29.6%)	7 (9.9%)	17 (23.9%)	12 (16.9%)	37 (52.1%)	5 (7.0%)
Paprika	25 (35.2%)	13 (18.4%)	8 (11.3%)	15 (21.1%)	10 (14.1%)	27 (38.0%)	16 (22.5%)	21 (29.6%)	7 (9.8%)
Parsley	20 (28.2%)	12 (19.6%)	8 (11.3%)	19 (26.8%)	12 (19.6%)	25 (35.2%)	11 (15.5%)	27 (38.0%)	8 (11.3%)
Rosemary	23 (32.4%)	15 (21.1%)	18 (25.4%)	12 (19.6%)	3 (4.2%)	28 (39.4%)	12 (16.9%)	27 (38.0%)	4 (5.6%)
Sage	30 (42.3%)	14 (19.7%)	11 (15.5%)	11 (15.5%)	5 (7.0%)	33 (46.4%)	13 (18.4%)	22 (31.0%)	3 (4.2%)
Thyme	26 (36.6%)	15 (21.1%)	14 (19.7%)	11 (15.5%)	5 (7.0%)	34 (48.0%)	14 (19.7%)	20 (28.1%)	3 (4.2%)
Turmeric	38 (53.5%)	11 (15.5%)	7 (9.9%)	8 (11.3%)	7 (9.9%)	40 (56.3%)	9 (12.7%)	14 (19.7%)	8 (11.3%)

### Association of spice and herb frequency and variety with diet quality, demographics, and health conditions

The Spearman correlation showed significant positive associations with overall diet quality scores and certain spices and herbs such as basil (r = 0.33; p = 0.006), cinnamon (r = 0.37; p = 0.002), ginger (r = 0.28; p = 0.02), paprika (r = 0.33; p = 0.006), rosemary (r = 0.24; p = 0.05), and turmeric (r = 0.27; p = 0.03). Similarly, positive associations were observed with whole grains, greens and beans, and total vegetables with basil, cilantro, cinnamon, cloves, coriander, cumin, ginger, mint, paprika, sage, and thyme. Seafood and plant proteins had positive associations with Cajun seasoning (r = 0.32; p = 0.006), chili pepper (r = 0.30; p = 0.01), cumin (r = 0.29; p = 0.02), and paprika (r = 0.29; p = 0.02). Total protein had positive associations with basil (r = 0.26; p = 0.04), cinnamon (r = 0.30; p = 0.01), and paprika (r = 0.26; p = 0.03). Negative associations were seen with refined grains and Adobo seasoning, black pepper, Cajun seasoning, chili pepper, cloves, curry seasoning, and Greek seasoning. There were positive associations with total variety of spice consumption once daily or more with greens and beans (r = 0.29; p = 0.02) and total vegetables (r = 0.25; p = 0.04) and negative association with refined grains (r = -0.28; p = 0.02). Positive associations were observed with total spice and herb variety consumed less than once daily with overall diet quality, greens and beans, total vegetables, whole fruits and total fruits, and total proteins as seen in Table 5.

**Table 5:** Spearman correlations with spice and herb frequency, spice and herb variety, and diet quality (n = 71).

	Overall diet quality	Whole grains	Refined grains	Greens & Beans	Total Vegetables	Whole fruit / Total fruit	Seafood & Plant proteins	Total proteins	Dairy
Adobo seasoning	0.00	-0.31*	0.17	0.24	0.24	-0.14	0.20	0.01	0.04
Basil	0.33**	-0.16	0.32**	0.40**	0.39**	0.03	0.21	0.26*	0.06
Black pepper	0.11	-0.30*	0.10	0.36**	0.26*	0.18	0.10	0.09	-0.04
Cajun seasoning	-0.06	-0.37**	0.01	0.27*	0.17	-0.09	0.32**	-0.08	0.08
Chili pepper	-0.02	-0.30*	0.02	0.42**	0.18	-0.06	0.30*	-0.05	0.08
Cilantro	0.16	-0.15	0.32**	0.26*	0.35**	-0.05	0.05	0.04	-0.01
Cinnamon	0.37**	-0.04	0.36**	0.38**	0.34**	0.04	0.22	0.30*	0.09
Cloves	0.11	-0.31*	0.27*	0.38**	0.32**	-0.09	0.13	-0.02	0.12
Coriander	0.21	-0.15	0.29*	0.26*	0.39**	-0.06	0.16	0.16	0.00
Cumin	0.23	-0.21	0.26*	0.39**	0.29*	0.04	0.29*	0.07	0.00
Curry leaf	0.15	-0.21	0.14	0.36**	0.31**	-0.15	0.26*	0.14	0.11
Curry seasoning	0.01	-0.28*	0.21	0.26*	0.16	-0.04	0.14	-0.02	0.03
Fennel	0.25*	-0.10	0.32**	0.36**	0.34**	-0.08	0.21	0.07	0.17
Garlic	0.08	-0.14	0.24*	-0.01	0.09	-0.06	-0.06	0.17	0.08
Ginger	0.28*	-0.17	0.38**	0.40**	0.28*	0.13	0.17	0.08	-0.01
Greek seasoning	-0.07	-0.32**	0.10	0.21	0.25*	0.00	0.11	-0.11	0.05
Italian seasoning	0.09	-0.15	0.08	0.17	0.22	-0.12	-0.03	0.12	0.26*
Mint	0.20	-0.11	0.32**	0.36**	0.33**	-0.03	0.10	0.01	-0.00
Oregano	0.22	-0.02	0.25*	0.31**	0.14	-0.06	0.09	0.14	0.10
Paprika	0.33**	-0.05	0.25*	0.33**	0.24*	0.10	0.29*	0.26*	-0.02
Parsley	0.21	-0.00	0.15	0.23	0.20	-0.09	0.10	0.29*	0.06
Rosemary	0.24*	0.03	0.23	0.25*	0.26*	0.00	0.03	0.24*	0.03
Sage	0.26*	0.01	0.26*	0.37**	0.34**	0.02	0.07	0.11	0.01
Thyme	0.30*	-0.01	0.30*	0.36**	0.28*	0.04	0.09	0.18	0.03
Turmeric	0.27*	-0.14	0.20	0.41**	0.39**	0.11	0.23	0.09	0.07
Average Spice and herb Frequency (daily)	0.20	0.19	-0.13	0.30*	0.23	-0.01	0.10	0.20	0.13
Spice and herb variety of consuming at least once daily or more	0.06	0.18	-0.28*	0.29*	0.25*	-0.32	0.16	0.04	0.05
Spice and herb variety of consuming less than once daily to once monthly	0.56**	0.29*	0.08	0.31**	0.40**	0.33**	0.06	0.35**	-0.14

Notes.

**. Correlation is significant at the 0.01 level (2-tailed);

*. Correlation is significant at the 0.05 level (2-tailed)

Focusing on associations with demographics and spice and herb intake, negative associations were observed with self-identifying as white with average spice and herb frequency consumption, total spice variety consumed more than once daily, turmeric, ginger, cumin, cinnamon, chili pepper, parsley; and stage of CKD with black pepper and parsley in Table 6.

**Table 6:** Spearman correlations with spice and herb frequency, spice and herb variety, and demographics (n = 71).

	Gender	Age	Race^a^	Ethnicity	CKD stage
Adobo seasoning	0.12	-0.25*	-0.23*	-0.21*	-0.05
Basil	0.01	-0.11	-0.04	-0.03	-0.08
Black pepper	-0.04	-0.12	0.05	0.09	-0.31**
Cajun seasoning	-0.01	-0.29**	-0.31**	0.10	-0.29**
Chili pepper	0.05	-0.23*	-0.28**	-0.12	-0.11
Cilantro	-0.01	-0.32**	-0.26*	-0.14	-0.24*
Cinnamon	0.11	0.09	-0.29**	0.07	-0.09
Cloves	0.05	-0.21*	-0.25*	0.10	-0.12
Coriander	0.01	-0.20	-0.31**	-0.18	-0.10
Cumin	-0.20	-0.16	-0.18	-0.14	-0.11
Curry leaf	0.10	-0.17	-0.26*	0.08	-0.07
Curry seasoning	-0.02	-0.14	-0.32**	0.12	0.05
Fennel	0.09	-0.21*	-0.25*	0.08	-0.23*
Garlic	0.14	-0.37**	-0.01	-0.04	-0.22*
Ginger	0.01	-0.04	-0.17	0.02	-0.10
Greek seasoning	0.01	-0.28*	-0.30**	0.09	-0.21*
Italian seasoning	0.18	-0.17	-0.22*	0.17	-0.04
Mint	-0.06	-0.18	-0.24*	0.11	0.00
Oregano	0.06	-0.20	0.03	-0.01	-0.20
Paprika	-0.07	-0.16	-0.20	0.14	-0.10
Parsley	0.09	-0.18	-0.23*	0.15	-0.27*
Rosemary	0.05	-0.08	-0.25*	0.04	-0.04
Sage	0.07	0.02	-0.18	0.13	-0.08
Thyme	-0.01	-0.05	-0.19	0.14	-0.16
Turmeric	0.05	0.07	-0.30**	0.11	0.01
Average Spice Frequency (daily)	0.09	-0.16	-0.34**	0.06	-0.18
Spice variety of consuming at least once daily or more	0.05	-0.19	-0.36**	0.16	-0.21
Spice variety of consuming less than once daily to once monthly	-0.09	0.10	-0.07	0.01	-0.13

Notes.

^a^. Race was condensed to white or not white due to participants who identified as non-white.

**. Correlation is significant at the 0.01 level (2-tailed);

*. Correlation is significant at the 0.05 level (2-tailed)

Negative associations were seen with certain health conditions like gastric reflux and chili pepper, and gastrointestinal issues and turmeric and cinnamon while positive associations were observed with total average spice and herb frequency consumption, total spice and herb variety consumed more than once daily, turmeric and ginger with cancer, and turmeric with heart disease in Table 7. From the sub-group analysis, no statistical differences were observed with early CKD stages (1–2) to late CKD stages (3–5) with spice frequency and quantity as seen in S1 Table.

**Table 7:** Spearman correlations with spice and herb frequency and health conditions (n = 71).

	Cancer	Depression	Diabetes	Diverticulosis / Diverticulitis	Gastric reflux	Heart disease	Hypertension	Irritable bowel	Liver issues	Nausea / Vomiting
Adobo seasoning	0.22*	0.01	-0.18	0.06	-0.10	-0.06	-0.25*	-0.21*	-0.18	-0.08
Basil	0.19	0.02	-0.05	0.04	-0.17	0.07	-0.12	-0.20	-0.11	-0.06
Black pepper	-0.08	0.01	0.16	0.05	-0.19	-0.17	-0.01	0.00	0.12	0.00
Cajun seasoning	0.18	0.02	-0.16	-0.03	-0.21*	-0.14	0.00	-0.01	-0.04	0.00
Chili pepper	0.12	0.09	-0.08	-0.12	-0.28**	0.00	-0.12	-0.21*	-0.21*	-0.08
Cilantro	0.24*	0.07	-0.21*	-0.02	-0.12	-0.09	-0.25*	-0.15	0.00	0.12
Cinnamon	0.23*	-0.03	0.14	0.07	-0.11	0.17	0.12	-0.25*	-0.05	-0.14
Cloves	0.28**	0.12	-0.15	0.01	-0.09	0.09	-0.09	-0.20*	-0.01	-0.01
Coriander	0.33**	-0.09	-0.15	-0.03	-0.10	0.16	-0.08	-0.19	-0.12	0.01
Cumin	0.23*	-0.05	-0.16	-0.13	-0.21*	0.09	-0.04	-0.15	-0.14	-0.08
Curry leaf	0.24*	0.04	-0.23*	0.03	-0.11	0.11	-0.23*	-0.11	-0.01	0.09
Curry seasoning	0.27*	0.13	-0.04	0.05	-0.08	0.06	-0.07	-0.12	-0.09	0.00
Fennel	0.38**	-0.05	0.02	0.01	-0.03	-0.02	-0.11	-0.24*	-0.08	-0.05
Garlic	0.09	0.05	-0.11	-0.05	0.20*	0.00	-0.18	0.09	0.05	0.17
Ginger	0.26*	0.07	0.03	0.16	-0.11	-0.03	-0.12	-0.11	-0.09	-0.04
Greek seasoning	0.21*	0.20*	-0.17	-0.11	0.07	-0.15	-0.25*	-0.15	0.05	-0.01
Italian seasoning	0.12	0.13	0.02	0.09	0.04	-0.01	-0.01	0.01	0.05	0.00
Mint	0.30**	0.00	0.06	0.01	0.08	0.02	-0.16	-0.25*	0.00	0.01
Oregano	0.15	-0.01	0.06	0.01	0.14	0.05	-0.10	-0.04	0.06	0.12
Paprika	0.20	0.04	0.10	0.05	-0.12	0.12	-0.04	-0.10	0.18	0.00
Parsley	0.22*	-0.06	-0.06	0.03	0.04	-0.04	-0.03	-0.06	0.04	0.13
Rosemary	0.21*	-0.13	-0.06	-0.03	0.05	0.16	0.07	-0.18	-0.04	-0.04
Sage	0.25*	-0.05	0.09	0.04	0.10	0.23*	0.07	-0.20*	0.06	0.02
Thyme	0.29**	-0.04	0.00	0.03	0.15	0.19	0.00	-0.14	0.14	0.11
Turmeric	0.32**	-0.07	0.18	-0.05	-0.05	0.28*	-0.02	-0.25*	-0.15	-0.10
Average Spice and herb Frequency (daily)	0.27*	0.02	0.03	-0.04	0.02	0.11	-0.13	-0.11	0.05	0.09
Spice and herb variety of consuming at least once daily or more	0.26*	0.00	0.03	-0.02	-0.06	0.01	-0.16	-0.13	0.07	0.07
Spice and herb variety of consuming less than once daily to once monthly	0.08	0.01	0.15	0.09	0.02	0.19	0.07	-0.14	-0.13	-0.10

Notes.

**. Correlation is significant at the 0.01 level (2-tailed);

*. Correlation is significant at the 0.05 level (2-tailed)

This study sought to determine frequency and quantity of spice and herb consumption and the association between diet quality and its food components, demographics, and health conditions with spice and herb frequency and variety of adults with CKD. Participants overall consumed at least 5 g of spices and herbs and black pepper and garlic more than once daily with other spices and herbs consumed less frequently. Positive associations were observed with spice and herb frequency of consumption and variety of consumption and overall diet quality and food groups. Further analysis showed associations with demographics such as self-identifying as white and conditions such as cancer, gastric reflux, and gastrointestinal issues with spice and herb frequency and variety.

It is recommended that adults with CKD follow a kidney friendly diet, which is focused on protein, potassium, phosphorus, and sodium [1,6,8]. In this study, the median diet quality of participants was considered in need of improvement (38.5 out of 60). The food groups with the lowest scores were whole grains and seafood and plant proteins with the highest scores in refined grains and total proteins. This aligns with other studies, in which diet quality scores and scores in certain food groups were similar of those with CKD [13,14]. These results may be attributed to recommendations to avoid or reduce consumption of foods high in potassium and phosphorus. Based on the actual CKD SFFQ, the foods consumed most frequently were raw fruits and non-starchy vegetables, baked or grilled chicken, and olive oil with limited consumption of fried foods and red meats. Even though beverages, aside from 100% fruit juice, were not considered in the overall diet quality score, participants consumed water, coffee, and tea daily and consumed sugar-sweetened beverages and products with alternative sweeteners on a weekly basis or less. It appears that participants were adhering to certain recommendations such as focusing on high-quality proteins like chicken and eggs and reducing consumption of red meats [1,27–29]. As participants indicated that beyond having CKD, they also had other chronic diseases such as heart disease and diabetes, which may have influenced dietary choices such as higher amounts of lean proteins and less products with added sugars and sodium.

Based on the results, minimal variety of spices and herbs were consumed more than once daily. Most of the spice and herb variety was consumed less than once daily with a quantity of 5g. This may have been related to health benefits, culture and demographics, taste, and dietary preferences. Garlic and black pepper were consumed, on average, one or more times daily with participants indicating that they used at least 5 g. This may have been related to the health benefits associated with these spices, although, that question was not asked. Furthermore, participants were not asked how they used these spices–prior to, during, or after the cooking/preparation method and if these spices/herbs were used during the cooking process the exact preparation method–boiling, steaming, etc. This may affect the antioxidant capacity and ability to improve kidney outcomes for those with CKD. A narrative review illustrated that garlic reduced the progression of CKD and inflammation and lipid levels in adults [21]. The authors rationalized that this improvement in biomarkers may have been related to the active compound, allicin, that is found within garlic. However, as the studies included within the review had differing forms of administering the garlic (i.e., whole, powder, supplement) with varying length, frequency, and quantity and preparation methods (length of cooking time and method used–boiling, steaming), it is unknown if the frequency and quantity of garlic that participants of this study consumed would positively impact their disease state. The bioavailability of allicin depends on its synthesis being activated through some form of damage to the cellular membrane, which would happen when the garlic clove is being cooked or through other processing methods used to activate this compound [27]). A study was conducted to determine the total phenolic and flavonoid content and antioxidant activity based on the solution, cooking time and temperature for garlic bulbs. Results showed cooking garlic bulbs in an aqueous solution for 60 minutes at a temperature of 150°C had the highest content of both total phenolic and flavonoids and highest antioxidant activity compared to the ethanol solution and other cooking times and temperatures [28]. For black pepper, the main active component is piperine that has many pharmacological effects such as cardioprotective, antihypertensive, and immunomodulatory [19,29]. Piperine is sensitive to heat and through various heating methods–boiling or use of pressure cooker, 16–34% can be lost [30,31]. This will impact the potency and ability of piperine on organs and other physiological functions. However, as no human studies have focused on black pepper for those with CKD, it is not known the frequency and quantity necessary to observe an improvement in kidney function. Other spices such as cinnamon, Italian seasoning, oregano, paprika, and parsley was used 2–6 times weekly. While spices such as black pepper and garlic possess distinctive flavors, others such as oregano and thyme are perceived to have milder flavors that may result in their potential health benefits being overlooked. As these spices and herbs were consumed less frequently, it is not known if participants were consuming these spices and herbs for any health benefits. Other studies have demonstrated that consuming at least 2.5 g of turmeric reduced inflammation markers for adults on dialysis [32] and consuming at least 50 g of chili daily in either dried or powdered reduced prevalence of CKD [33]. Even though this current study did not associate specific spice/herb intake with kidney outcomes nor with the use of these spices during the cooking process, further research should focus on these aspects to determine the impact on kidney outcomes.

The least frequently used spices and herbs were Adobo seasoning, coriander, curry leaf and fennel. According to a cross-sectional study that explored the consumption of spices among adults (n = 703), results showed that the most consumed spices were black pepper, garlic, and cinnamon. Researchers indicated that demographics (predominately white, non-Hispanic) and knowledge contributed to this low consumption and variety of spices [26]. As in this study, 78.9% of participants self-identified as white, non-Hispanic this may have contributed to the limited varieties of spices and herbs consumed. Even though this survey did not include this question, the limited spices and herbs consumed may have been due to limited knowledge and application for these spices and herbs.

Positive associations were observed with black pepper and cinnamon with certain food groups such as greens and beans, total vegetables, whole grains, and total protein. Spices and herbs are frequently utilized while cooking to enhance the sensory characteristics of dishes [27]. This corresponds to the associations observed between spice frequency and variety–whether once daily or less than once daily, overall diet quality, and the food groups in this study. For example, Cajun seasoning was positively associated with seafood and plant proteins. Cajun seasoning is a blend of various spices such as paprika, salt, garlic powder, black pepper and oregano and is typically used to marinate and season seafood dishes like gumbo and jambalaya [28]. The use of spices may have been more associated with the taste and preference when used on certain dishes than possibly the cultural aspect. Wen and colleagues had similar results in their cross-sectional study among adults (n = 474,015) and associations with chili consumption and dietary habits. Participants who consumed more spices (i.e., chili paste, powder) had higher intakes of poultry and vegetables, snacks, and deep-fried foods. The researchers concluded that since food establishments use chili powder for deep-fried food and other products, it may have led to the higher consumption of these food items due to taste preference [18]. Another cross-sectional study among adults (n = 1042) illustrated that there were positive associations with the use of certain spices such as garlic, basil, mint, and oregano with vegetables [34]. This is similar findings to this study, which possibly participants were utilizing more spices with vegetables to enhance the taste and flavor of the vegetables. In this study, participants focused on the spices and herbs they added to the foods as opposed to the spices/herbs that may have been found in the product. For example, if a participant consumed commercially prepared soup, they were not asked to include the spices/herbs from that product, but instead if they added additional spices and/or herbs to that prepared product. Participants completed a food frequency questionnaire as opposed to 24-hour recalls, thus there was an inability to calculate the exact amount of spice usage and to know if the spice was consumed in conjunction with one another or independently. Further research must be completed to analyze the correlation of taste preferences and the frequency and variety of spice consumption.

Another rationale for the use of certain spices may have been due to the chronic condition the participant had in addition to CKD. For example, the use of turmeric was positively associated with participants who had heart disease while ginger intake was positively associated with participants who had cancer. Even though human clinical trials are limited with different spices, those that have focused on certain pungent spices such as garlic, ginger, turmeric, black pepper have shown improvements in health conditions [19,21,23,30–32]. As demonstrated in reviews, turmeric leads to reduction in inflammation, blood pressure, oxidative stress and others due to its active compound curcumin [33,35]. Furthermore, several reviews have indicated the benefits of consuming ginger to prevent cancer or reduce side effects associated with cancer treatments, which is largely due to its active compounds 6-gingerol and 6-shogaol [32,36,37]. As with the studies mentioned for garlic and black pepper, inconsistencies in the form, quantity, and frequency of administering these spices were unable to demonstrate that the quantity and frequency consumed in this study aided in alleviating symptoms or reduced the progression these conditions as those questions were not included on the survey. It is noteworthy that spices that may contribute to discomfort such as chili pepper were negatively associated with certain disease states such as irritable bowel syndrome and gastric reflux. This is likely due to the discomfort caused by consuming these spices either through the esophagus or intestine [38].

Moreover, spices are generally recommended for adults with CKD for flavor enhancement of cuisines when reducing sodium [39,40]. Even though 66.2% of participants within this study reported having hypertension, no positive correlations were observed with frequency of spice consumption or variety of spices consumed, yet more with negative associations, which likely were related to avoiding seasonings/spices with high salt content. Adobo seasoning was negatively associated with hypertension, which may be contributed to the salt content within this seasoning as 1.25 g may have more than 300 mg of sodium [41]. At least in the US, recommendations for adults with hypertension are to reduce sodium content to 2300 mg and informed about seasonings and spices that contain sodium, it may contribute to the reduced intake of this seasoning.

There were some limitations to this study that may have influenced results. Participants self-reported their dietary intake and spice frequency and quantity over the past 30-days. The instrument did not provide metric weights or pictorial images to aid in the quantity. Therefore, it is unknown if the quantity registered was accurate. Limited questions focused on knowledge or awareness of what spices they consumed in meals when they ate outside the home or even how the spices/herbs were used–prior to, during or after the cooking/preparation methods. For example, curry leaf is commonly used to season Indian dishes [26]. It is possible participants consumed these dishes but were unaware of the spices in them. The sample size of 71 was a limitation to this study as the results cannot be generalized to the average CKD population. Even though various recruitment protocols took place over several months, limited individuals responded, which may have been related to the timing of the study or else that it took place online. Furthermore, participants self-reported their CKD stage due to this survey was administered to the US population, thus obtaining medical charts and blood samples were not part of this study. Even though the researchers conducted their recruitment in kidney disease specific areas, it is still not known if their self-reported stage would have been confirmed with other measurements such as estimated Glomerular Filtration Rate.

## Conclusion

This study showed associations among spice and herb frequency and variety with diet quality and its food components in adults with various stages of CKD. Consumption of spices such as basil, cinnamon, ginger, paprika, rosemary, and turmeric showed a significant positive association with overall diet quality scores. Additionally, higher variety of spices showed a positive association with diet quality scores. This information could be beneficial for additional recommendations in potentially improving kidney disease progression. Further research should focus on spices used in flavoring foods to evaluate the on kidney function for adults with CKD.

## Supporting information

S1 Table Mann-Whitney U tests for spice frequency and quantity of CKD stages (n = 54). (DOCX)
