# Correction: Associations between diet quality, demographics, health conditions and spice and herb intake of adults with chronic kidney disease

**DOI:** 10.1371/journal.pone.0329835

**Published:** 2025-08-05

**Authors:** Emma Hammer, Sofia Acevedo, Jeanette Mary Andrade

The first author’s name is spelled incorrectly. The correct name is: Emma Hammer. The correct citation is: Hammer E, Acevedo S, Andrade JM (2024) Associations between diet quality, demographics, health conditions and spice and herb intake of adults with chronic kidney disease. PLoS ONE 19(3): e0298386. https://doi.org/10.1371/journal.pone.0298386.
