# Supplementary material for: Associations between diet quality, demographics, health conditions and spice and herb intake of adults with chronic kidney disease
Source: PLoS One. 2024 Mar 7;19(3):e0298386. doi: 10.1371/journal.pone.0298386 (PMC10919673; doi:10.1371/journal.pone.0298386)
Supplement: S1 Table — (DOCX) [file pone.0298386.s001.docx]

**S1 Table. Mann-Whitney U tests for spice frequency and quantity of CKD stages (n=54).**

| **Item** | **All CKD stages (n=69)*** | **Early CKD Stages (n=11)** | **Later CKD Stages (n=43)** | **Z** | **p-value** |
| --- | --- | --- | --- | --- | --- |
|  | Median (range) | Median (range) | Median (range) |  |  |
| Average spice frequency (daily) | 0.22 (0.00 - 6.00) | 0.17 (0.05 – 1.14) | 0.20 (0.00 – 1.45) | -0.25 | 0.81 |
| Average spice quantity (g) | 0.48 (0.00 - 1.50) | 0.48 (0.08 – 1.16) | 0.42 (0.00 – 0.73) | -1.08 | 0.28 |
| Notes: *All CKD stages includes stages 1 – 5 and do not know | | | | | |
